# Temperature outweighs diet in shaping developmental performance in two cricket species via growth delays and physiological limits

**DOI:** 10.1242/jeb.251472

**Published:** 2025-12-12

**Authors:** Émile Vadboncoeur, Marie-Hélène Deschamps, Susan M. Bertram, Heath A. MacMillan

**Affiliations:** ^1^ Department of Biology, Carleton University, Ottawa, ON, Canada, K1S 5B6; ^2^ Département des Sciences Animales, Faculté des Sciences de l'Alimentation et de l'Agriculture, Pavillon Paul-Comtois, Université Laval, Québec, QC, Canada, G1V 0A6

**Keywords:** Environmental stressor, Nutrition, Development, Growth, Trade-offs, Parametrization

## Abstract

Understanding how chronic environmental stressors shape animal development is essential for predicting ecological responses and optimizing rearing systems. This perspective complements the use of short-term tolerance assays, which overlook the cumulative effects of sustained stress. Temperature and nutrition affect key life-history traits such as growth, development rate and survival. While both factors have been widely studied, their relative impacts are not clearly defined. We investigated how constant temperature (26–41°C) and dietary protein-to-carbohydrate (P:C) ratio (0.15–2.18) influence development in two cricket species, *Acheta domesticus* and *Gryllodes sigillatus*. Growth trajectories were modelled using a unified logistic equation to estimate asymptotic mass and maximum growth rate, thereby capturing the growth trajectory in a simplified and interpretable way, enabling comparisons across treatments. Asymptotic mass was combined with developmental rate and survival to calculate a composite metric of developmental performance. Developmental performance peaked at 35°C but fell at thermal extremes as a result of delayed development (in cold) or reduced mass and survival (in heat). Diet had more modest effects, as performance was stable across most P:C ratios, and only declined at extreme imbalances. Notably, the performance cost of the most unbalanced diets was comparable to a 4–5°C shift from thermal optimum. Our results demonstrate that temperature, more than diet, drives variation in developmental performance during *ad libitum* feeding. This integrative framework provides a robust approach to quantify environmental sensitivity, define performance limits and guide us toward the mechanisms underlying those limits and/or performance trade-offs.

## Teaser

**Summary:** Temperature more strongly influences growth trajectories and developmental performance of crickets than diet, because of delayed development and reduced survival rate at thermal extremes.

## INTRODUCTION

Understanding how chronic environmental stressors shape insect development is critical for predicting ecological responses and optimizing mass rearing systems. Traits such as development, growth and survival are influenced by both temperature and diet (two of the most widely studied factors; Clissold and Simpson, 2015; Hardison et al., 2021; Johnson et al., 1992; Raynal et al., 2022; Sissener et al., 2021). Collectively, these traits govern the pace and relative success of completing development from embryo to mature adult with broad applications in conservation, pest control and mass rearing. The relative influence of temperature and diet on each of these traits, however, remains unclear.

While dietary manipulations are frequently tested over long periods or the full course of development, short-term exposures to temperature are often used to estimate optimal temperatures and upper and lower thermal limits, particularly through metrics such as critical thermal limits. Although convenient, these acute assays have been increasingly criticized for their limited ecological relevance (Desforges et al., 2023; Kingsolver and Umbanhowar, 2018; Leong et al., 2022; Ørsted et al., 2022). In contrast to diet, which is rarely thought of as an acute stress, high temperatures may appear tolerable in short-term trials despite ultimately impairing survival rate, growth or development (Abarca et al., 2024; Jørgensen et al., 2021; Rezende et al., 2014, 2020). Survivor bias can therefore obscure biologically meaningful thresholds and hinder comparisons across species or treatments with differing physiological sensitivities. Chronic exposure experiments, which assess multiple traits over extended periods, offer a more integrative understanding of thermal stress (Abarca et al., 2024; Kong et al., 2025a; Magozzi and Calosi, 2015). By quantifying survival rate, developmental time and mass under constant temperature conditions, we can better identify sublethal thermal limits and compare their effects with those of dietary imbalance, contextualizing trade-offs across environmental gradients throughout development.

**Fig. 1.**
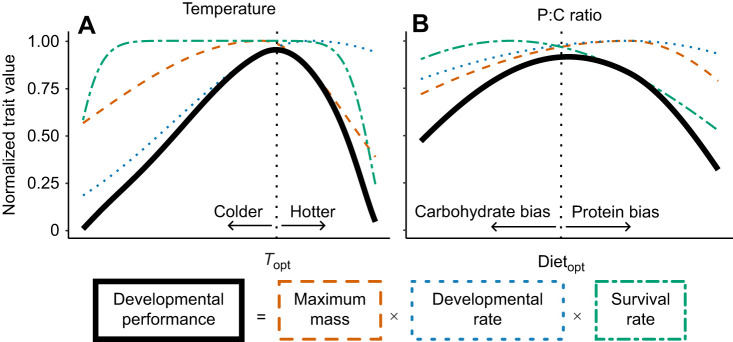
**Conceptual framework illustrating how temperature and diet influence key life-history traits and how these traits combine into a composite metric of developmental performance.** Effects of temperature (A) and protein-to-carbohydrate (P:C) ratio on two normalized life-history traits: maximum mass (orange dashed line) and developmental rate (blue dotted line), and survival rate (green dot-dashed line), which multiply together to form a developmental performance metric (solid black line). The existing literature guides the shape of these predictive curves for insects with a specific focus on Orthoptera when appropriate (see Table S1). In A, survival rate remains high over a broad thermal range before declining sharply at upper and lower limits; developmental rate increases with temperature up to an optimum, then stabilizes or declines; and maximum mass tends to peak at intermediate to high temperatures in orthopterans, decreasing at both extremes, with a steeper decline at high temperatures. Developmental performance peaks at an optimal temperature (*T*_opt_), where the combined product of traits is maximized. At higher temperatures, performance declines steeply as a result of reduced survival rate and mass, while at cooler temperatures, it declines more gradually, primarily because of slower development despite high survival rate and mass. Thermal limits in insects are generally well defined by temperatures at which animals cannot survive over short to medium time frames. In B, survival rate peaks on carbohydrate-rich diets and declines with increasing protein content; developmental rate increases with protein content, peaking at balanced or protein-biased P:C ratios; and maximum mass follows a similar trend, reaching its highest values near balanced or protein-biased diets and decreasing under both protein- and carbohydrate-dominated conditions. Developmental performance peaks at an optimal macronutrient ratio (Diet_opt_), where survival rate, growth and development are simultaneously maximized. At carbohydrate-biased extremes, performance declines as a result of slower development and smaller body size, while at high protein levels, elevated mortality constrains performance. Unlike temperature limits, dietary critical limits are less abrupt, but extreme macronutrient imbalances can still restrict developmental success. By integrating multiple traits, this metric allows direct comparison of how different environmental parameters shape overall developmental outcomes and reveals which exerts greater influence across the tested range. We hypothesize that the amplitude of variation in developmental performance across the dietary gradient will be smaller than that across the thermal gradient, reflecting the more stringent physiological constraints imposed by extreme temperatures. We also hypothesize that the effect of temperature will be constant, while the effect of diet will be more stable with effects towards dietary extremes.

Insect traits typically vary across environmental gradients, with traits maximized in intermediate environments and trending lower toward environmental extremes. Temperature effects on animal traits have long been a research focus, and thermal performance curves have been well documented across insect taxa and traits (Alruiz et al., 2023; Magara et al., 2024; Rebaudo and Rabhi, 2018). Survival probability, for example, remains high across a broad range of moderate temperatures but declines steeply near species-specific critical thermal limits (Fig. 1A; Abarca et al., 2024; Régnière et al., 2012). Developmental rates follow a typical thermal performance pattern, increasing with temperature up to a maximum value and then dropping sharply (Fig. 1A; Gibert and De Jong, 2001; Lamb and Gerber, 1985; Morales-Ramos et al., 2024). Mass at adulthood is not a trait that thermal performance curves can accurately describe (Angilletta, 2009). For many ectotherms, body size at adulthood follows the temperature–size rule, where animals grow larger at lower temperatures (Atkinson, 1994). However, some taxa grow largest at intermediate or higher temperatures, including orthopterans, such as crickets (Fig. 1A; Kong et al., 2025b; Whitman, 2008).

In contrast to temperature, the effects of diet on performance are more difficult to generalize because of variation in ingredient composition. However, a standardized approach for studying diet effects on performance in insects involves manipulating the ratio of protein-to-carbohydrate (P:C) (Raubenheimer and Simpson, 1999; Simpson and Raubenheimer, 1995). As protein content increases, survival rate tends to decrease (Fig. 1B; Bouchebti et al., 2022; Dussutour and Simpson, 2012; Lee, 2015; Lee et al., 2008a; Muzzatti et al., 2024; Nicholls et al., 2021), while developmental time is typically shortest on balanced or slightly protein-biased diets (Fig. 1B; Kim et al., 2020a,b; Lee, 2015; Muzzatti et al., 2024; Roeder and Behmer, 2014). Adult mass is similarly maximized under balanced to slightly protein-biased conditions (Fig. 1B; Jang and Lee, 2018; Kaewtapee et al., 2024; Muzzatti et al., 2024; Roeder and Behmer, 2014). Performance declines at the extremes of the P:C ratio spectrum (approaching 1:0 or 0:1; Muzzatti et al., 2024; Roeder and Behmer, 2014). But, unlike temperature, clear physiological critical limits for P:C ratios are not well defined, in part because of the impressive ability of insects to regulate intake and compensate for imbalanced diets (Behmer, 2009).

Traditionally, traits such as developmental time, maximum mass and survival rate have been examined independently to assess the effects of environmental conditions on organismal performance. However, evaluating these traits in isolation can obscure underlying trade-offs and interactions. For example, larger body sizes achieved through prolonged development may be disadvantageous in season-limited environments (Angilletta et al., 2004; Nufio et al., 2025). To address this, we adopted a formula proposed by Muzzatti et al. (2024) that integrates these three traits into a single measure of individualized yield: the amount of biomass produced per unit time, conditional on survival. While Muzzatti et al. (2024) refer to this metric as yield, reflecting its relevance to farming contexts, we refer to it here as developmental performance, as it captures multiple traits during the developmental period. In our revised version of the metric, we also normalize mass and developmental rate to the mean of the control group to facilitate comparison between species and sexes that reach different adult sizes. Plotting developmental performance across a range of parameter values (Fig. 1) allows clearer identification of optima and limits, and enables more comprehensive comparisons between parameters such as temperature and diet. This approach addresses calls for more holistic metrics of performance that reflect ecological fitness (Abarca et al., 2024).

Understanding how environmental conditions affect insect development is key not only for predicting population dynamics in ecological contexts (Camacho et al., 2024; Overgaard et al., 2014) but also for improving outcomes in the context of insect mass rearing for farming (Kong et al., 2025b; Li et al., 2023; Muzzatti et al., 2024), pest management (Johnson et al., 1992; Nyamukondiwa et al., 2013; Quispe-Tarqui et al., 2021) and mitigating disease spread (Lee et al., 2008b; Wojda, 2017). Thermal sensitivity of insect traits is frequently studied to anticipate range shifts, optimize rearing conditions or inform control strategies (Kong et al., 2025b; Pinkert et al., 2025). While insect diets are also of interest for these purposes (Clissold and Simpson, 2015; Muzzatti et al., 2024), diet effects are rarely evaluated across multiple temperatures in a systematic way. Some studies have explored temperature–diet interactions, demonstrating that ambient temperature can shift the optimal P:C ratio for growth and development (Clissold et al., 2013; Lazarević et al., 2023; Lee and Roh, 2010), and that macronutrient regulation and digestive physiology respond to temperature variation (Lemoine and Shantz, 2016; Rho and Lee, 2025). However, comprehensive studies that compare the two variables remain scarce (Min et al., 2020), especially across full developmental trajectories or in a comparative framework. Identifying which environmental axes have the greatest influence on developmental performance could guide more targeted decision making.

In this study, we examined two cricket species, *Gryllodes sigillatus* and *Acheta domesticus*, to assess how a range of temperatures and dietary P:C ratios affect developmental time, growth and survival rate, and a composite metric of developmental performance that integrates all three traits. Our design allows us to quantify the relative magnitude of environmental effects on each trait and combine them in a single developmental metric. We hypothesize that the amplitude of variation in developmental performance across the dietary gradient will be smaller than that across the thermal gradient, reflecting the more stringent physiological constraints imposed by extreme temperatures. We also hypothesize that the effect of temperature will be constant, while the effect of diet will be more stable, with greater effects towards dietary extremes.

## MATERIALS AND METHODS

### Cricket rearing

Our *Gryllodes sigillatus* (Walker 1869) colony originated from eggs from Entomo Farms (Norwood, ON, Canada) and is maintained at Carleton University (Ottawa, ON, Canada), where 20 families are kept for laboratory use. One week after reaching adulthood, the crickets lay eggs in peat moss over 2 days and are then incubated until hatching. Eggs of *Acheta domesticus* (Linnaeus 1758) came from Aspire Food Group (London, ON, Canada). Adults laid eggs in peat moss overnight, after which the moss containing the eggs was transferred into an insulated container with heat packs and shipped to Carleton University. Eggs arrived within 24 h of being laid, and were then returned to an incubator to complete development. All eggs were incubated at 32°C under a 14 h light:10 h dark photoperiod in moistened peat moss. The experiment started one day after hatching (11 and 9 days for *G. sigillatus* and *A. domesticus*, respectively). One-day post-hatching crickets were placed in individual condiment containers (96 ml, 7 cm diameter×3 cm depth; Fig. S1) that contained a piece of egg carton for shelter. Water was provided via a 1.5 ml microcentrifuge tube sealed with dental cotton, allowing crickets to drink. Feed was provided *ad libitum* in a small plastic ink cap (1.7 cm diameter×1.4 cm depth) glued to a 50 mm Petri dish. The feed dishes and water tubes were swapped every 2 and 3 days, respectively. The crickets were kept on a 14 h light:10 h dark photoperiod at a relative humidity ranging from 65% to 85% for all incubators. Temperature and humidity were recorded every 30 min using an environmental logger (Inkbird, Shenzhen, China).

### Experimental treatments

The experimental design was established to facilitate the comparison between the effects of temperature and diet by linking treatments using a control (32°C; 0.83 P:C ratio) for both species. Each treatment combination included 30 individual crickets per species (unless mentioned otherwise below), resulting in a total sample size of 700 individuals.

Crickets of both species were reared on a control diet (balanced diet of 0.83 P:C ratio) at different temperatures (26, 29, 32, 35, 38, 41°C). The control diet was selected because it approximates the intake target observed in self-selection trials for *G. sigillatus* (1.05 P:C ratio; Muzzatti et al., 2024). While no specific intake target has been documented for *A. domesticus*, orthopterans generally select balanced diets (Behmer, 2009). All temperature treatments were conducted using a customized array of mini-incubators (Vevor Inc., Shanghai, China).

Crickets of both species were reared on different diets at a constant temperature of 32°C. For *G. sigillatus*, the temperature was chosen because it lies at the lower end of the thermal range that maximizes most life-history traits (Kong et al., 2025b). For *A. domesticus*, 32°C is slightly higher than the 30°C proposed by Clifford and Woodring (1990), but still within the optimal range (28–35°C; Clifford et al., 1977).

Seven (0.15, 0.22, 0.45, 0.83, 1.36, 1.75 and 2.18; P:C ratio) and five (0.15, 0.22, 0.45, 0.83 and 1.75; P:C ratio) experimental diets were tested in *G. sigillatus* and *A. domesticus,* respectively. For *G. sigillatus*, diets from 0.45 to 2.18 were divided evenly across four mini-incubators, with six individuals per treatment per incubator. The two most carbohydrate-biased diets (0.15 and 0.22) were tested in a follow-up trial, housed in three additional incubators. Each of these incubators contained ten individuals per new diet, plus five individuals from the 0.45 and 0.83 groups to ensure consistency with the earlier trial. For *A. domesticus*, treatments were conducted in a large incubator (Thermo Fisher Scientific Inc., Waltham, MA, USA). *Acheta domesticus* reared under the control (32°C; 0.83 P:C ratio) treatment were reared in both mini and large incubators with 20 crickets per incubator type to ensure the rearing environment did not affect the traits we measured.

### Experimental diets

Oligidic diets were formulated using ingredients from a nearby feed mill (Campbellford Mill, Campbellford, ON, Canada) to better reflect natural or applied feeding conditions. Proportions of ingredients in the experimental diets were chosen to achieve different P:C ratios (Table 1). We opted for this approach over the geometric framework (Raubenheimer and Simpson, 1999; Simpson and Raubenheimer, 1995) as the geometric framework lacks a straightforward means of comparison with temperature, a continuous variable that cannot be diluted in the same way as diet. To standardize particle size and prevent selective feeding, all ingredients were sifted to remove particles larger than 1 mm, and any remaining larger particles were ground using a mortar and pestle until they passed through a 1 mm mesh.

**Table 1.** Formulation and proximal composition of oligidic diets having different protein-to-carbohydrate (P:C) ratios

	P:C ratio						
	0.15	0.22	0.45	0.83	1.36	1.75	2.18
Ingredients (%)							
Corn	86	73.8	47	28.8	10	3	2
Soymeal	5	10	33	29	22	13	3
Canola meal	2	5	5	5	5	5	5
Corn gluten	4.3	8	10.8	25	50.8	62.8	62.6
Fishmeal	0.5	1	2	10	10	14	25
Hog pro mix	2.2	2.2	2.2	2.2	2.2	2.2	2.2
Composition (%, dry basis)							
Crude proteins	12.5	16.8	26.9	39.6	51.4	56.8	59.7
Carbohydrates	80.3	75.0	62.0	49.0	37.6	32.4	27.4
Crude lipids	1.8	1.8	2.3	2.8	2.7	2.7	3.8
Crude fibres	2.4	3.0	3.4	2.8	2.5	2.0	1.8
Ash	3.0	3.4	5.3	5.8	6.0	6.2	7.3
Dry matter (%)	88.5	88.7	89.1	90.4	90.7	91.0	91.4
Energy (kcal g^−1^)	3.9	3.9	4.0	4.3	4.5	4.7	4.7

Hog pro mix is a mixture of minerals and vitamins for swine feed produced by Bio-Ag Consultants and distributors (Wellesley, ON, Canada).

Proximal analyses were conducted for all diets (Table 1). Ash content was measured by incinerating diet samples at 600°C for 13 h according to the Association of Official Analytical Collaboration (AOAC) Official Method 942.05 (Thiex et al., 2012) using a Lindberg/Blue M 1100 C Box Furnace (ThermoScientific, Burlington, ON, Canada). Proteins were determined using the Dumas method (high-temperature combustion) in accordance with AOAC Official Method 992.23, using a FP828p analyser (LECO Corporation, Mississauga, ON, Canada). Total lipids were extracted with diethyl ether using an XT15 extractor (ANKOM Technology, Macedon, NY, USA) according to the American Oil Chemists' Society (AOCS) Standard Procedure Am 5-04. Crude fibre content was measured by sequential digestion with 0.1275 mol l^−1^ H_2_SO_4_ and 0.313 mol l^−1^ NaOH using ANKOM filter bags, following AOCS Standard Procedure Ba-6a-05, and analysed with an ANKOM 200 fibre analyser (ANKOM Technology). Total carbohydrate content was estimated by subtracting the sum of measured ash, protein, lipid and fibre values from 100%. Energy content was determined via bomb calorimetry using a 6400 Automatic Isoperibol Calorimeter (Parr Instrument Co., Moline, IL, USA), following the manufacturer's instructions.

All diets used whole or defatted feed ingredients (e.g. corn, corn gluten, soymeal, canola meal, fish meal) to vary P:C ratio without purified nutrients. As a result, other nutritional components such as lipid, ash and energy content also varied among diets. Crude lipid varied over a small range (∼2–4%) that has previously been shown not to influence life-history traits in *A. domesticus* and other terrestrial insects (Oonincx et al., 2020; Starčević et al., 2017). Ash content was slightly higher in the protein-rich diets than in the carbohydrate-rich diets. However, animals had constant access to water, and the feed was supplemented with vitamins and minerals, minimizing the risk of micronutrient imbalance. Energy content increased with protein content, as diets were not diluted to equalize energy. This was a deliberate choice to preserve a similar amount of combined protein and carbohydrate (∼87–92%) as P:C studies often aim for (Dussutour and Simpson, 2012; Le Gall and Behmer, 2014; Lee et al., 2008b). Some degree of compensatory feeding to adjust caloric intake is to be expected, but this small difference is unlikely to cause differences that would impact our conclusions (Jang and Lee, 2018).

### Data collection

Three times a week, the survival status was noted, and the position of each container was randomized to avoid positional effects. Every 7 days, surviving individuals were weighed using an analytical scale (Mettler Toledo AB135-S). Sex (when discernible at antepenultimate instar) and instar (based on moult counts) were recorded according to Kong et al. (2025a). The experiments were stopped at 6 weeks (42 days) as this encompassed the developmental period of the two species.

### Modelling individual growth and development

Data from insects are increasingly modelled to observe the effects of diet (Kaewtapee et al., 2024; Sripontan et al., 2020) or temperature (Grunert et al., 2015) on growth. Here, we fitted models to the growth and development trajectories of each individual cricket and compared the resulting parameters within species across treatments to better understand how diet and temperature affect these traits. To evaluate how mass changed over time, we modelled individual growth curves using three-parameter sigmoid unified logistic regressions (Tjørve and Tjørve, 2017). Unified logistic models were selected for their superior fit and interpretability across treatments when compared (*R*^2^ values and visual inspection of residuals) with other commonly used sigmoid models describing insect growth (Grunert et al., 2015; Kaewtapee et al., 2024; Sripontan et al., 2020): unified (U)-Gompertz (*n*=555, mean *R*^2^=0.995; where *n* is the number of individuals the indicated model converged for, with mean *R*^2^ for the model of each of those individuals), U-Bertalanffy (*n*=476, mean *R*^2^=0.963) and U-Richards (*n*=505, mean *R*^2^=0.907). As the Gompertz and Bertalanffy models tended to overestimate asymptotes, the unified logistic regression (*n*=579, mean *R*^2^=0.994) was chosen for its consistent convergence and superior fit, especially in estimating asymptotic mass.

**Fig. 2.**
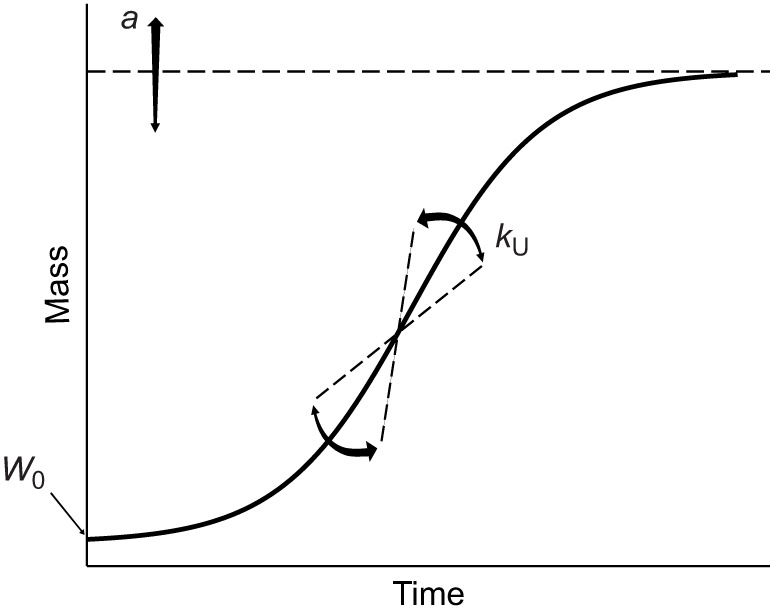
**Schematic diagram of a cricket growth curve to illustrate the three parameters of the unified logistic model (Eqn 1).** The asymptote (*a*) represents the estimated maximum body mass at the end of development. The intercept (*W*_0_) reflects the body mass at hatching. The parameter *k*_U_ indicates the relative maximum growth rate, describing how quickly the curve approaches the asymptote. Together, these parameters characterize the full trajectory of growth from hatch to maturity.

Unified logistic models have two forms: one uses the inflection point, while the other uses the *y*-intercept to anchor the curve. As our study measured growth from hatching, we used the intercept-based form to anchor the curve to the starting mass (i.e. hatching mass) of each cricket. We therefore used the unified logistic formula as follows:
(1)

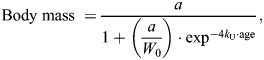
where body mass is in mg, *a* is the asymptotic mass (i.e. maximum body mass), *W*₀ is the body mass at hatch (*y*-intercept), and *k*_U_ is the relative growth rate, which controls how quickly the curve approaches the asymptote (i.e. *k*_U_=0 is flat, *k*_U_=∞ is vertical). Fig. 2 illustrates how each parameter influences the growth curve.

**Fig. 3.**
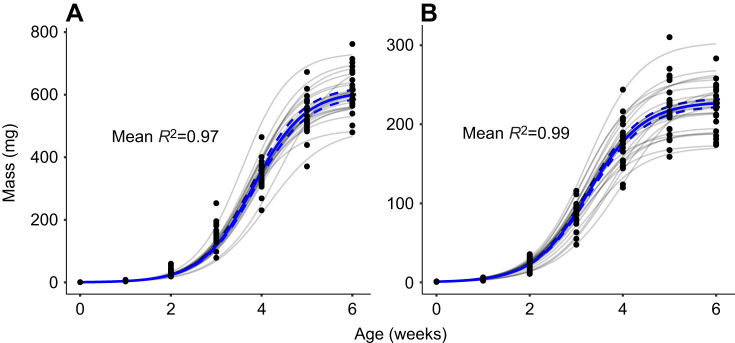
**Examples of individual and average growth trajectories of *Acheta domesticus* and *Gryllodes sigillatus* on the control diet treatment.** (A) Female *A. domesticus* (*n*=21) and male *G. sigillatus* (*n*=23) growth trajectories, representing every individual from those groups surviving throughout the experiment on the control treatment (32°C on a 0.83 P:C ratio diet). Black points represent weekly body mass measurements for each cricket. Grey lines show unified logistic model fits for each cricket, while the bold blue line represents the mean fitted curve with shaded 95% confidence interval. Mean *R*^2^ values indicate model fit across individuals within each group.

Developmental time is typically measured as the number of days until reaching the final moult or a specified pre-adult instar (Magara et al., 2024; Morales-Ramos et al., 2024; Muzzatti et al., 2024). In this study, instar stage was recorded weekly, which did not allow us to precisely determine the day of adult emergence. Instead, we estimated developmental rate by fitting a linear regression to weekly instar data for each individual:
(2)

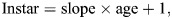
where the slope represents the rate of instar progression, and the intercept was fixed at 1, corresponding to the first instar at hatch. Only individuals surviving to at least week 5 were included in this analysis. An exponential model (*n*=478, mean *R*^2^=0.985) was also tested, but the linear model (*n*=575, mean *R*^2^=0.962) was preferred for its simplicity and convergence across individuals. Model fits were strong overall, with *R*^2^ values >0.95 for growth and >0.85 for development. Notably, this technique preserves variance in individual growth and development trajectories but also compounds errors from individual regressions when comparing mean parameters for the different experimental groups. With consistently strong model fits (high *R*^2^ values), we are confident that this error is minimized and that the parameters accurately represent the growth and development of our experimental animals. Examples of unified logistic growth curves are shown in Fig. 3 and Fig. S2.

### Developmental performance

Developmental performance was calculated using the yield metric described by Muzzatti et al. (2024), which integrates survival rate, developmental rate and adult body mass into a single value for every individual. The metric was adapted as follows:
(3)

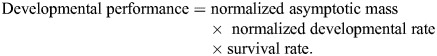
The asymptotic mass was derived from the unified logistic growth model (parameter *a*; see ‘Modelling individual growth and development’, above). The developmental rate is the slope of the rate of instar progression through time (see ‘Modelling individual growth and development’, above). Both asymptotic mass and developmental rate are normalized to control conditions (32°C; 0.83 P:C ratio) so all parameters are on a similar scale to improve comparability between species. Survival rate was calculated per treatment group and applied uniformly to all individuals within that group. It was defined as the proportion of individuals alive on day 42 relative to initial number of individuals in that group. This value, specific to each species and treatment group, was not normalized, as it already falls between 0 and 1 and reflects a biologically meaningful component of development.

### Modelling parameters

To model the effects of temperature and diet on developmental rate and performance, we fitted thermal performance curves using models from the ‘rTPC’ package in R. For temperature-dependent traits, several models were tested, and the best-fitting models were selected based on the Akaike information criterion (AIC). For diet-dependent traits, only a quadratic model was tested, as used in nutritional geometry (Jensen et al., 2015; Lee et al., 2008a). For each trait, the model with the lowest AIC was visually inspected to ensure it captured the general shape of the response. The best-fitting model was then used to generate a continuous curve describing how each trait varied across the temperature or diet.

### Statistical analysis

All analyses used R 4.3.3 (R Core Team 2024; https://www.r-project.org/). All figure data are reported as means±sem. Given the goal of the current study was to compare the thermal and dietary effects, species were not compared statistically; our analysis compared the different treatments (temperature and diet) and sexes. To fit non-linear models, we used the *nls_multstart* function from the ‘nls.multstart’ package to extract parameter estimates. All parameters were compared with the control group of crickets held at 32°C and 0.83 P:C ratio. Survival data were analysed using a Cox proportional hazards model with Firth's penalized maximum likelihood estimation, implemented via the *coxphf* function in the ‘survival’ package, with species and treatment as predictors. Extracted growth and development parameters, as well as developmental performance, were analysed using generalized linear models with a Gamma distribution, incorporating treatment, sex and their interaction as fixed effects. The *stepAIC* function from the ‘MASS’ package was used to identify the most parsimonious models. The selected models can be found in Table 2. Their outputs and information to interpret them can be found in Table S2–S12. Every generalized linear model was examined to ensure that the residuals were independent and free of outliers. *Post hoc* pairwise contrasts of each treatment against the control were obtained with the ‘emmeans’ package using the ‘trt.vs.ctrl’ method, with Holm corrections applied to adjust for multiple testing.

**Table 2.** Statistical models used to evaluate trait responses in *Acheta domesticus* and *Gryllodes sigillatus*

Parameter	Predictors	
	*A. domesticus*	*G. sigillatus*
Asymptotic mass (*a*)	Treatment+Sex	Treatment+Sex
Relative maximum growth rate (*k*_U_)	Treatment×Sex	Treatment×Sex
Developmental rate	Treatment+Sex	Treatment×Sex
Survival	Treatment	Treatment
Developmental performance	Treatment	Treatment

Models were selected using stepwise model selection based on Akaike's information criterion (stepAIC), starting from a full factorial model including treatment and sex. For survival and developmental performance, sex was not included as a predictor. Sex was not identifiable for many of the non-survivors, and normalization of the parameters for developmental performance removed the significance of sex.

## RESULTS

### Developmental rate: temperature-driven, diet stable

**Fig. 4.**
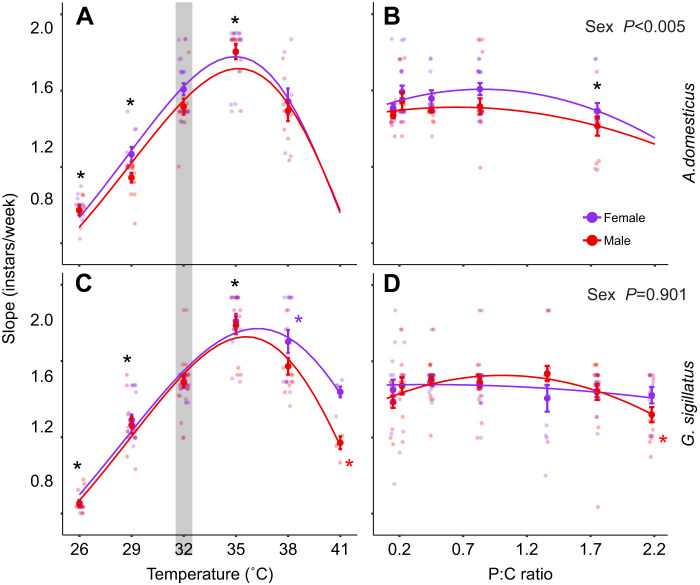
**Developmental rate for male (red) and female (purple) *A. domesticus* (top) and *G. sigillatus* (bottom) under different temperature and dietary P:C ratios.** Developmental rate was calculated as the slope (instars per week) of a linear regression fitted to weekly instar data. (A,C) Developmental rates across temperatures (26, 29, 32, 35, 38 and 41°C); (B,D) rates across diets varying in P:C ratio (0.15, 0.22, 0.45, 0.83, 1.36, 1.75 and 2.18). Each point represents an individual; lines show model predictions. The shaded region indicates the control condition (32°C and 0.83 P:C ratio diet). Asterisks denote significant differences (*P*<0.05) from the control (black for both sexes or appropriate colour if sex interactions) based on *post hoc* pairwise contrasts with Holm corrections for multiple testing.

Developmental rate, measured as the slope of instar progression over time, was strongly influenced by temperature. Both *A. domesticus* and *G. sigillatus* developed faster at 35°C, with rates reaching approximately 1.8 instars per week, but declined at both cooler and warmer temperatures (Fig. 4A,C). In contrast to temperature, diet had minimal effects on developmental rate. The most protein-biased diet slowed the developmental rate of both sexes in *A. domesticus* (*t*=3.14, *P*<0.01; Fig. 4B), but only for males in *G. sigillatus* (*t*=3.58, *P*<0.003; Fig. 4D).

### Growth trajectories

#### Overall effects of temperature and diet on growth

**Fig. 5.**
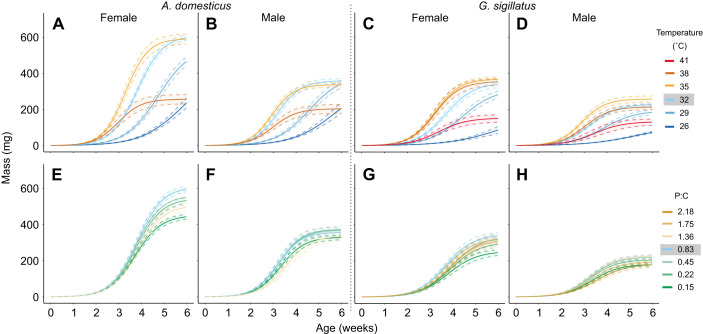
**Modelled growth trajectories of female and male *A. domesticus* (left) and *G. sigillatus* (right) reared under different temperature and dietary treatments.** (A–D) Mass over time across six temperatures (26, 29, 32, 35, 38 and 41°C). (E–H) Effects of seven P:C ratios (0.15, 0.22, 0.45, 0.83, 1.36, 1.75 and 2.18) on growth at a constant temperature of 32°C. Solid lines represent logistic regression fits; shaded ribbons indicate standard error. Female and male data are shown in the left and right columns of each species panel, respectively.

**Fig. 6.**
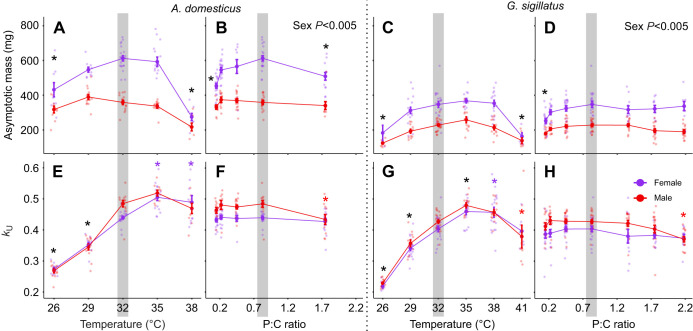
**Unified logistic model parameters describing growth trajectories in male (red) and female (purple) *A. domesticus* (left) and *G. sigillatus* (right).** (A–D) Asymptotic mass and (E–H) relative maximum growth rate (*k*_U_), with effects shown across temperature (A,C,E,G) and dietary P:C ratio (B,D,F,H). Each point represents an individual cricket reared from 1 day post-hatch to day 42 under a single treatment. The shaded region represents the control condition (32°C and 0.83 P:C ratio diet). Asterisks indicate a significant difference (*P*<0.05) from the control (black for both sexes or appropriate colour if sex interactions) based on *post hoc* pairwise contrasts with Holm corrections for multiple testing. Asymptote refers to the modelled adult body mass, and *k*_U_ represents the rate at which mass increases toward the asymptote.

Temperature and diet shaped overall growth trajectories of both species by influencing asymptotic mass and relative maximum growth rate (*k*_U_; Figs 5 and 6). Temperatures above or below the thermal optimum reduced *k*_U_, flattening the growth curve and delaying its progression toward the asymptote, which itself also declined. For example, female *A. domesticus* (Fig. 5A) reared at 32°C reached 99% of their asymptotic mass (612±15 mg) in 6.4±0.1 weeks, whereas those reared at 26°C reached 99% of a lower asymptote (432±41 mg) in 10.0±0.2 weeks. At 38°C, female *A. domesticus* reached 99% of their final mass more quickly (5.4±0.3 weeks), but the asymptote was less than half the control value (257±26 mg). Similar temperature-related trade-offs were observed across both sexes and species, with 41°C being the temperature reducing the asymptote at the higher end for *G. sigillatus* (Fig. 5C,D).

In contrast, the effects of diet on growth trajectories were comparatively modest. For instance, female *A. domesticus* (Fig. 5E) fed the lowest P:C ratio diet exhibited a reduced asymptotic mass (−35%), but reached 99% of it in the same amount of time as those on the control diet (6.4±0.1 weeks). Likewise, female *G. sigillatus* (Fig. 5G) fed the lowest P:C ratio diet reached an asymptote 27% lower than the control, without a notable delay in timing. Other diets caused less than a 20% reduction in asymptotic mass and did not significantly affect timing. In summary, extreme diets primarily reduced final mass with minimal impact on growth duration, while suboptimal temperatures altered both the mass attained and the speed at which it was achieved.

#### Parametrization of growth

Newly hatched *A. domesticus* nymphs had an average mass of 0.74±0.09 mg (*n*=310), consistent with the initial mass (*W*_0_) estimated by the unified logistic model (0.74±0.09 mg). By the final measurement day, control females reached 615±15 mg, significantly larger than control males (354±14 mg; *t*=13.79, *P*<0.001). The model-predicted asymptotic masses closely matched these values, validating the logistic model, with estimated asymptotes of 612±15 mg for females and 359±13 mg for males (Fig. 6A–D). The relative maximum growth rate (*k*_U_) was 0.44±0.01 for females and 0.49±0.01 for males (Fig. 6E–H). These sex differences were statistically significant for both asymptotic mass (*F*=326.27, *P*<0.001) and *k*_U_ (*F*=19.79, *P*<0.001) (Fig. 6).

High and low temperatures, as well as the most biased diets, significantly reduced asymptotic mass. *k*_U_ was affected by temperature: higher temperatures increased *k*_U_, while lower temperatures reduced it. Sex differences in *k*_U_ were temperature dependent, as shown by a significant interaction between sex and temperature (*F*=2.73, *P*=0.007). These differences were primarily due to females increasing growth rates compared with control, while males did not, and to males having reduced growth rates under the most protein-biased diet, while females did not.

Newly hatched *G. sigillatus* had an average mass of 0.85±0.17 mg (*n*=390), closely matching the starting mass (*W*_0_) estimated by the unified logistic model (0.86±0.16 mg). At the end of the study, control females reached 331±18 mg and males 223±6 mg. These final masses aligned with the model's estimated asymptotes of 347±18 mg for females and 229±7 mg for males. Despite larger hatchling mass, *G. sigillatus* adults were smaller than *A. domesticus*, indicating divergent growth strategies. The relative maximum growth rate (*k*_U_) was 0.40±0.01 for females and 0.43±0.01 for males. As with *A. domesticus*, these sex differences in both asymptotic mass (*F*=19.76, *P*<0.001) and *k*_U_ (*F*=22.677, *P*<0.001) were statistically significant. All reported treatment differences are relative to control crickets reared at 32°C and fed a 0.83 P:C ratio diet (Fig. 6).

Asymptotic mass of *G. sigillatus* declined to 144±24 mg at 26°C, 142±12 mg at 41°C, and 220±10 mg on the lowest-protein diet. Similar to *A. domesticus*, *k*_U_ increased with temperature until 35°C and returned to baseline at 38°C for males and 41°C for females. Males reared at 41°C and fed the most protein-biased diet both had values of *k*_U_ below baseline, indicating that *G. sigillatus* males are more sensitive to nutritional and thermal stress than females.

### Survival

**Fig. 7.**
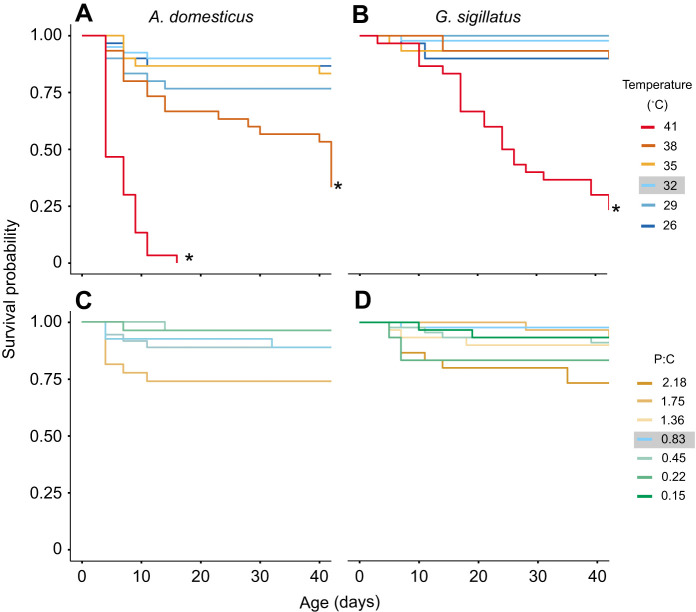
**Survival probability of *A. domesticus* (left) and *G. sigillatus* (right) under different temperature and dietary P:C ratios.** (A,B) Survival at six constant temperatures (26, 29, 32, 35, 38 and 41°C). (C,D) Survival on seven experimental diets varying in P:C ratio (0.15, 0.22, 0.45, 0.83, 1.36, 1.75 and 2.18). Asterisks indicate a significant difference (*P*<0.05) compared with the control group (32°C, 0.83 P:C ratio diet; grey) based on *post hoc* pairwise contrasts with Dunnett's corrections for multiple testing. Each group began with 30 individuals, except for the 0.83 and 0.45 P:C ratio diet treatments at 32°C, which began with 45 individuals.

The survival rates in the control group (32°C and 0.83 P:C ratio) were 87.5% for *A. domesticus* and 97.8% for *G. sigillatus* (Fig. 7). Survival was only significantly reduced at the highest temperature tested. For *A. domesticus*, survival at 41°C dropped to 0% by day 16. This rapid mortality suggests a critical upper thermal limit near 41°C for *A. domesticus*. By day 42, survival was 33% at 38°C and 77% on the 1.75 P:C ratio diet. *Gryllodes sigillatus* exhibited greater thermal tolerance, maintaining 27% survival at 41°C on day 42, and survival on the 0.22 and 2.18 P:C ratio diets was 83% and 73%, respectively. Although the most extreme diet reduced survival rates, *post hoc* Dunnett's tests revealed no statistically significant differences compared with the control.

### Developmental performance: integrating growth, survival and timing

**Fig. 8.**
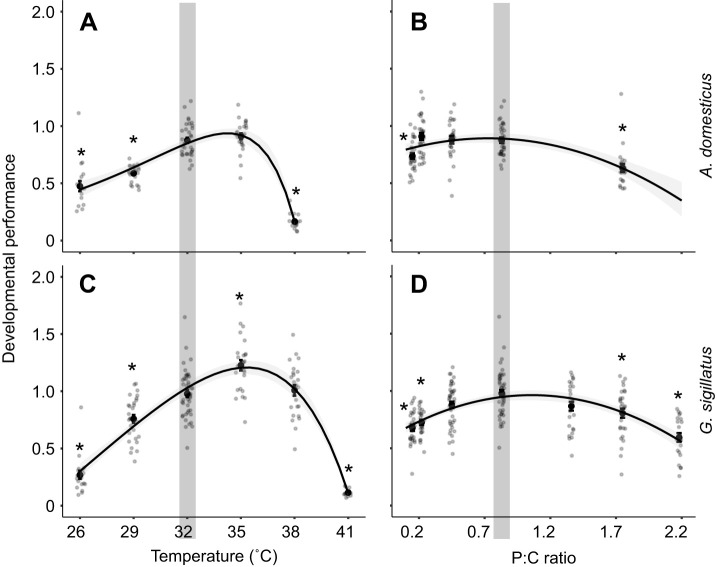
**Developmental performance of *A. domesticus* (top) and *G. sigillatus* (bottom) reared from egg to adulthood under different temperatures and dietary P:C ratios.** Each point represents an individual cricket. Developmental performance was calculated by multiplying the ratio of asymptotic mass to mean asymptotic mass of the control group by the survival probability of its treatment group and dividing by the number of weeks required to reach the 8th instar. Crickets were reared at six constant temperatures (26, 29, 32, 35, 38 and 41°C; A,C) and on seven experimental diets varying in P:C ratio (0.15, 0.22, 0.45, 0.83, 1.36, 1.75 and 2.18; B,D). The shaded region indicates the control condition (32°C and 0.83 P:C ratio diet). Asterisks denote significant differences (*P*<0.05) from the control based on *post hoc* pairwise contrasts with Holm corrections for multiple testing.

Developmental performance was more strongly impacted by temperature than by diet. Performance peaked at 35°C and declined at both thermal extremes, as a consequence of different traits (Fig. 8), highlighting the asymmetric nature of thermal stress: low temperatures delayed development, while high temperatures reduced mass and survival rate. Dietary effects on developmental performance were more modest, with reductions observed only at the most extreme P:C ratios; specifically, the highest protein and carbohydrate levels. These findings reinforce the conclusion that temperature exerts a stronger influence on developmental performance than diet. Normalizing mass and growth rate to the mean of the control group rendered sex a non-significant influence on the developmental performance of both species, and we therefore excluded it from the analysis (*A. domesticus*: *t*=−0.059, *P*=0.95; *G. sigillatus: t*=0.787, *P*=0.43). For *A. domesticus*, developmental performance peaked at 35°C with the 0.83 P:C ratio diet (0.91±0.03) and remained equivalent to control levels at 32°C on the same diet (0.88±0.02). From those temperature and dietary peaks, performance declined by 48% at 26°C (0.47±0.04) and 82% at 38°C (0.17±0.01). Developmental performance only dropped by 19% (0.73±0.03) on the 0.15 P:C ratio diet and by 30% (0.63±0.04) on the 1.75 P:C ratio diet. Thus, the maximum change in developmental performance due to temperature exceeded that due to diet. For *G. sigillatus*, developmental performance peaked at 35°C with the 0.83 P:C ratio diet (1.26±0.05). For the dietary range, control crickets at 32°C with the 0.83 P:C ratio diet had the highest developmental performance (0.98±0.03). Performance declined by 91% to 0.11±0.01 at the highest temperature and by 79% to 0.27±0.03 at the lowest temperature; performance declined by 36% to 0.68±0.03 on the most carbohydrate-biased diet and by 40% to 0.59±0.04 on the most protein-biased diet. Across both species, the largest diet-driven decline in developmental performance was roughly equivalent to only a 4–5°C decrease from the thermal optimum.

## DISCUSSION

Here, we found that both temperature and diet influenced developmental outcomes in two species of crickets. Developmental performance peaked at 35°C in both species and declined at both thermal extremes, but for different reasons: low temperatures delayed development while high temperatures reduced mass and survival rate. Temperature exerted a more continuous influence on time-dependent traits such as developmental and growth rates. In contrast, diet effects were comparatively stable and only affected traits at the most extreme P:C ratios. These findings highlight the importance of understanding chronic effects: while insects can maintain near-optimal traits across intermediate conditions, extreme environments impose disproportionate trade-offs. Prior studies examining the interactive or comparative effects of temperature and diet found similar effects: the impact of temperature is typically strong and continuous, while dietary effects are strong at the edge of the P:C ratio range in holometabolous insects (Kim et al., 2020a; Lee and Roh, 2010; Min et al., 2020; Petersen et al., 2000). By quantifying these chronic, multi-trait responses, our study refines our understanding of how environmental conditions shape growth and development.

Our study complements existing nutritional and physiological frameworks used to study insect responses to environmental stress. Research on dietary effects in insects has relied mainly on controlled nutritional frameworks, each with strengths but also important limitations in scope. The geometric framework of nutrition has been especially influential for identifying nutrient-specific limitations and behavioural intake targets (Jang and Lee, 2018; Lee et al., 2008b; Raubenheimer and Simpson, 1999). However, it is methodologically demanding and limits experimental scope, especially when integrating additional factors such as temperature. Studies aiming to assess the interactive effects of temperature and diet often use fixed-ratio diets (Kim et al., 2020a; Lee and Roh, 2010; Rho and Lee, 2017). These studies, however, have had restricted developmental windows, either within a single larval instar or within a period of adulthood (Lee and Roh, 2010; Lee et al., 2015; Petersen et al., 2000; Rho and Lee, 2017). While these designs offer valuable mechanistic insights, they risk obscuring the cumulative impacts of chronic environmental conditions, effects that may appear tolerable within one stage but would impair trait levels in previous or subsequent stages. By following crickets from hatching to adulthood, our study captures the consequences of chronic exposure and enables a direct comparison of dietary and thermal effects across the whole developmental trajectory.

The shape of the thermal performance curves for the different traits measured in both *A. domesticus* and *G. sigillatus* showed similar patterns. We found parallel steep declines in the developmental performance curves at high temperatures that were driven by reduced growth and mortality. Similar to other studies, lower temperatures resulted in smaller adults but did not drive mortality (Abarca et al., 2024; Kong et al., 2025b; Lamb and Gerber, 1985; Magara et al., 2024). Our findings suggest temperatures below 26°C should be used to test the effects of chronic cold stress on these two species. Our findings align with previous work showing that thermal performance curves for rate-based traits are typically skewed, with sharp declines at upper thermal limits (Rebaudo and Rabhi, 2018; Shi and Ge, 2010). In both species, developmental rate and asymptotic mass peaked at intermediate to high temperatures and declined at thermal extremes, consistent with this pattern. Although asymptotic mass is not a rate-based trait (Angilletta, 2009), the final size of an insect is determined by the rate of biomass accumulation and the duration of that growth period. As a result, the temperature dependence of asymptotic mass likely reflects the combined effects temperature on growth rate and development duration (Forster and Hirst, 2012). Unlike many ectotherms that follow the temperature–size rule (Atkinson, 1994), orthopterans such as crickets tend to reach maximum size at intermediate to warm temperatures (Kong et al., 2025b; Magara et al., 2024; Tu et al., 2012; Whitman, 2008), suggesting the thermal sensitivities of growth and development are closely aligned in this group (Forster and Hirst, 2012).

The range of P:C ratio values we tested in this study had a comparatively limited effect on developing crickets. This was evident across all measured traits, including survival rate, growth and development (Figs 4–7). Similar results have been reported in other herbivorous insects, where traits are maintained despite variation in dietary macronutrient balance (Deans et al., 2015, 2022; Lee et al., 2006; Muzzatti et al., 2024). Insects can mitigate suboptimal nutrient intake through a combination of behavioural and physiological strategies. While behavioural compensation, such as selecting alternative foods to balance intake, is common (Behmer, 2009), our crickets were restricted to a single diet. Crickets might have behaviourally compensated for this by increasing their food consumption, but this necessarily results in overconsumption of the non-limiting nutrient, which can come with metabolic or toxic costs (Behmer, 2009), discussed further below.

We argue that high temperatures induce high inefficiency realized over a short developmental period, while low temperatures induce modest inefficiency that extends over a prolonged period. These two scenarios culminate in similarly diminished growth outcomes. Both temperature extremes reduced asymptotic mass, but only low temperatures delayed the time required to reach adult mass. The highest temperatures survived (38°C for *A. domesticus* and 41°C for *G. sigillatus*) led to dramatic decreases in asymptotic mass but only modest reductions in relative maximum growth rate. In contrast, cooler temperatures reduced both relative maximum growth rate and final body size. Metabolic rate increases with temperature (Lachenicht et al., 2010; Roe et al., 1980), and so do growth rates, up to an optimal point beyond which performance declines (Bjørge et al., 2018). At these high temperatures, the efficiency with which energy is converted to mass is low, and animals tend to mature smaller (Bjørge et al., 2018), suggesting an inability to put resources towards growth because of increased maintenance costs associated with high temperature (González-Tokman et al., 2020). Lower temperatures similarly induce low energy conversion efficiency, associated with reduced growth rates (Bjørge et al., 2018), possibly indicating similar increased physiological costs, but likely for different reasons. Low temperatures reduce metabolic rate (Lachenicht et al., 2010; Roe et al., 1980), but also slow developmental processes (Booth and Kiddell, 2007; Magara et al., 2024), thereby extending the duration over which maintenance costs accumulate. Neither metabolic rate nor development time scales linearly with temperature, so the mismatch between energy availability and demand is greater further from the thermal optimum (Marshall et al., 2020). These patterns align with the broader framework of energetic allocation trade-offs (Mauritsson and Jonsson, 2023; Zera and Harshman, 2001), which propose that organisms balance limited energetic resources among competing demands, typically growth, maintenance, reproduction and survival rate. At thermal extremes, resources are disproportionately reallocated toward maintenance and stress mitigation rather than somatic growth, leading to reduced asymptotic mass. Such trade-offs are central to life-history theory and have been observed in diverse ectotherms, including fish (Audzijonyte and Richards, 2018) and insects (Boggs, 2009), under environmental stress.

A similar accumulation of inefficiencies may explain the reduction in asymptotic mass under the most unbalanced diets. Crickets fed low-protein diets must consume more food to meet protein requirements (Simpson and Abisgold, 1985), potentially increasing the metabolic costs of feeding, digestion and nutrient processing (Deans and Hutchison, 2023). Excess carbohydrate intake has also been linked to ‘wastage respiration’, an increase in CO_2_ production that does not contribute to growth (Zanotto et al., 1997) or account for energy cost of relying more on lipid metabolism (Talal et al., 2021). In addition, carbohydrate-rich diets promote lipid accumulation in insects (Clark et al., 2014; Lee, 2015; Lee et al., 2015), which may advance the onset of moulting through size-sensing mechanisms (Nijhout and Callier, 2015). Therefore, the resulting individuals would be smaller if moulting is triggered before sufficient protein reserves are acquired to support maximum post-moult size.

High-protein diets may also reduce final mass, but through different costs. Surplus dietary protein is catabolized via gluconeogenesis (Zanotto et al., 1993), leading to nitrogenous waste excreted primarily as uric acid in insects (Weihrauch and O'Donnell, 2021). Because uric acid excretion is an active, energetically costly process, diets rich in protein may lead to energy being spent on excretion that could otherwise be allocated to growth, limiting maximum body size.

These patterns highlight how asymptotic mass and relative growth rate respond to different physiological constraints across thermal and dietary gradients. While the mechanisms driving reduced growth under extreme conditions likely differ, ranging from temperature-dependent metabolic inefficiencies to nutrient-specific processing costs, our modelling framework provides a powerful tool to disentangle their effects. By isolating growth rate and asymptote as independent parameters and reconstructing full growth trajectories, this approach allows researchers to infer the relative contributions of developmental timing, energy conversion efficiency, nutrient processing or any other possible mechanism to observed size outcomes. As such, growth modelling summarizes complex biological responses in a simplified and interpretable way and offers a pathway to develop and test mechanistic hypotheses about how environmental stressors reshape energy allocation strategies. This capacity makes it broadly applicable to both ecological research and applied efforts in insect rearing and trait optimization.

### Conclusion

Our results demonstrate that temperature exerts a stronger influence than diet on insect developmental performance. Performance peaked at 35°C in both species and declined at cooler and warmer temperatures, due to delayed development, and elevated mortality and reduced mass, respectively. Diet composition had comparatively modest effects, with performance maintained across a broad range of P:C ratios and only declining under the most unbalanced conditions.

By parameterizing growth trajectories and integrating developmental rate, maximum mass and survival into a unified performance metric, we propose a framework for assessing environmental sensitivity to different parameters. This approach may be particularly useful for comparing species with different life-history strategies or ecological niches and captures the developmental consequences of sustained environmental stress, providing a complementary lens to acute stress tolerance assays (Abarca et al., 2024; Kingsolver and Umbanhowar, 2018). Our results emphasize the value of multi-trait metrics in environmental research and offer a robust framework for future studies on environmental stress and life-history evolution.

## Supplementary Material

10.1242/jexbio.251472_sup1 Supplementary information
