# Supplementary material for: Temperature outweighs diet in shaping developmental performance in two cricket species via growth delays and physiological limits
Source: J Exp Biol. 2025 Dec 12;228(24):jeb251472. doi: 10.1242/jeb.251472 (PMC12752487; doi:10.1242/jeb.251472)
Supplement: Supplementary information [file jexbio-228-251472-s1.pdf]

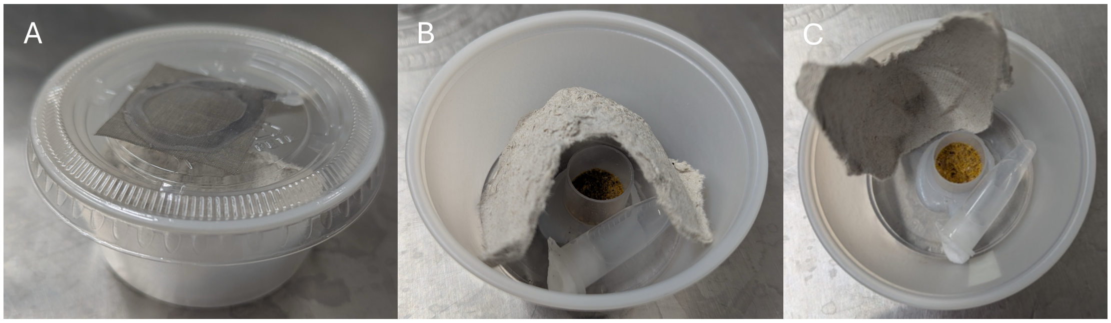

Fig. S1. Rearing setup for individual crickets. (A) Closed 96 mL condiment container (7 cm diameter × 3 cm depth) with a ventilated plastic lid. (B) Interior view of the container showing a section of egg carton used as a shelter, a 1.5 mL microcentrifuge tube filled with water and sealed with dental cotton, and a small plastic ink cap (1.7 cm diameter × 1.4 cm depth) containing feed, fixed to a 50 mm Petri dish. (C) Shelter removed to show positioning of feed and water sources. Crickets were housed individually in these containers throughout the experiment.

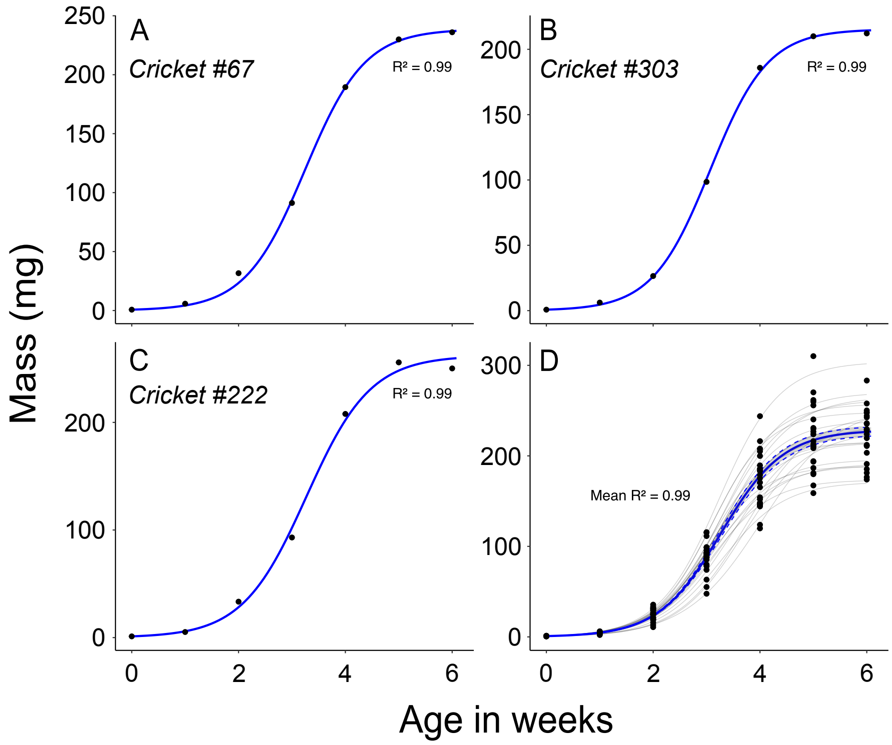

Fig. S2. Representative examples of individual cricket growth modelled using **Equation 1**. Panels A–C show three randomly selected *G. sigillatus* males reared at 32 °C on a 0.83 protein-to-carbohydrate (P:C) diet, with body mass (mg) measured weekly over six weeks. Panel D shows growth trajectories for all individuals in this treatment group (black points), with fitted unified logistic curves overlaid (gray lines). Blue lines in panels A to C represent the model fit. Blue line in panel D represents a model made with the mean ± standard error of all crickets from the group. R^2 values are reported for each individual model, with a mean R^2 across the group, indicating strong model performance.

Journal of Experimental Biology • Supplementary information

Table S1. References supporting the conceptual framework in **Figure 1**, illustrating the typical response curves of insect life-history traits (survival, developmental rate, and adult mass) to variation in temperature and dietary protein-to-carbohydrate (P:C) ratio. Each reference is accompanied by a brief description of the relevant pattern it documents.

**Reference**	**Summary Description**
Abarca et al., 2024	Survival declines sharply near critical thermal limits in insects multiple *Lepidoptera* species.
Régnière et al., 2012	Survival high across wide range of temperature and rapidly drops off at both ends of the range in *Choristoneura fumiferana* and *Bactrocera cucurbitae*.
Gibert and De Jong, 2001	Developmental rate increases with temperature, peaks close to higher thermal limit and then either maintains or decreases slightly in four *Drosophila* species.
Lamb and Gerber, 1985	Survival high across wide range of temperature and rapidly drops off at both ends of the range. Developmental rate increases with temperature, peaks close to higher thermal limit and is then maintained in *Entomoscelis americana*.
Morales-Ramos et al., 2024	Developmental rate increases with temperature, peaks close to higher thermal limit and then decreases rapidly in *Tenebrio molitor*.
Atkinson, 1994	Introduces the temperature-size rule in ectotherms. Most ectotherms, including many insect groups, will reach adulthood with larger mass at lower temperature.
Whitman, 2008	Orthopterans (including crickets) deviate from the temperature-size rule and grow largest at intermediate to high temperature.
Kong et al., 2025	Crickets grow largest at intermediate or high temperatures in *Gryllodes sigillatus*.
Dussutour and Simpson, 2012	Survival decreases with high protein intake in *Lasius niger*.
Lee, 2015	Survival decreases with high protein intake. Adult body weight increases with protein content in *Drosophila melanogaster*.
Lee et al., 2008	High protein reduces survival in *Drosophila melanogaster*.
Bouchebti et al., 2022	High protein reduces survival in *Blaptica dubia*.
Nicholls et al., 2021	High protein reduces survival in *Apis mellifera*.
Kim et al., 2020	Fastest development and adult mass on balanced to protein-biased diets. Lifespan maximized with low-protein diets in different strains of *Drosophila melanogaster*.
Muzzatti et al., 2024	Developmental rate and mass at adulthood increased with protein content in feed while survival decreased in *Gryllodes sigillatus*.
Roeder and Behmer, 2014	Development and mass maximized with moderate protein bias, while survival is maximized under carbohydrate-biased feeds in *Heliothis virescens*.
Kaewtapee et al., 2024	Adult mass is highest under balanced P:C in *Gryllus bimaculatus*.
Jang & Lee 2018	High developmental rate with protein-biased diets in *Drosophila melanogaster*.

Journal of Experimental Biology • Supplementary information

Table S2. Treatment numbers and their corresponding temperature and diet (protein:carbohydrate ratio, P:C).

Treatment	Temperature (˚C)	Diet - P:C
1	26	0.83
2	29	0.83
3	32	0.45
4	32	0.83
5	32	1.36
6	32	1.75
7	32	2.18
8	35	0.83
9	38	0.83
10	41	0.83
11	32	0.22
12	32	0.15

Table S3. Post-hoc comparisons using Dunnett's test to evaluate the effect of treatment on survival in *Acheta domesticus*, based on a Firth penalized Cox proportional hazards model. Treatment 4 served as the control group. Reported values include coefficient estimates (Coef.), standard errors (SE), z-values (t ratio), and p-values (Pr(>|t|)).

Dunnett Post-hoc Test (vs. Control)

Contrast	Estimate	SE	t ratio	Pr(>ltl)
Treatment 1 - Treatment 4	0.303	0.689	0.44	0.999
Treatment 2 - Treatment 4	0.884	0.616	1.44	0.597
Treatment 3 - Treatment 4	0.031	0.736	0.04	1.000
Treatment 6 - Treatment 4	0.912	0.616	1.48	0.564
Treatment 8 - Treatment 4	0.507	0.656	0.77	0.972
Treatment 9 - Treatment 4	1.966	0.538	3.65	0.002 **
Treatment 10 - Treatment 4	3.184	0.536	5.94	1.981e-08 ***
Treatment 11 - Treatment 4	-0.867	0.974	-0.89	0.938
Treatment 12 - Treatment 4	-0.857	0.974	-0.88	0.942

Signif. codes: 0 '***' 0.001 '**' 0.01 '*' 0.05 '.' 0.1 ' ' 1

Journal of Experimental Biology • Supplementary information

Table S4. Post-hoc comparisons using Dunnett's test to evaluate the effect of treatment on survival in *Gryllodes sigillatus*, based on a Firth penalized Cox proportional hazards model. Treatment 4 served as the control group. Reported values include coefficient estimates (Coef.), standard errors (SE), z-values (t ratio), and p-values (Pr(>|t|)).

Dunnett Post-hoc Test (vs. Control)

Contrast	Estimate	SE	t ratio	Pr(>ltl)
Treatment 1 - Treatment 4	1.305	1.028	1.27	0.667
Treatment 2 - Treatment 4	-0.710	1.720	-0.41	0.999
Treatment 3 - Treatment 4	1.131	0.993	1.14	0.764
Treatment 5 - Treatment 4	1.308	1.028	1.27	0.665
Treatment 6 - Treatment 4	0.907	1.088	0.83	0.939
Treatment 7 - Treatment 4	2.291	0.933	2.46	0.079 .
Treatment 8 - Treatment 4	0.956	1.088	0.88	0.921
Treatment 9 - Treatment 4	1.273	1.028	1.24	0.691
Treatment 10 - Treatment 4	3.485	0.888	3.92	6.581e-04 ***
Treatment 11 - Treatment 4	1.832	0.970	1.89	0.264
Treatment 12 - Treatment 4	0.937	1.088	0.86	0.928

Signif. codes: 0 '***' 0.001 '**' 0.01 '*' 0.05 '.' 0.1 ' ' 1

Table S5. Generalized linear model results evaluating the effects of treatment and sex on asymptotic mass in *Acheta domesticus*. ANOVA table (A) showing the significance of treatment and sex on asymptotic mass using a Gamma-distributed GLM with a log link. Pairwise post-hoc comparisons (B) between each treatment and the control (Treatment 4) based on estimated marginal means (EMMs), using treatment-vs-control contrasts and Holm correction for multiple testing. Reported values include coefficient estimates (Estimate), standard errors (SE), degrees of freedom (df), t-ratios, and p-values (Pr(>|t|)).

A. ANOVA Table for Asymptotic mass (a)

Effect	Df	Deviance	Resid. Df	Resid. Dev	F value	Pr(>F)
Treatment	8	8.720	215	14.996	40.358	< 2e-16 ***
Sex	1	8.812	214	6.184	326.273	< 2e-16 ***

Signif. codes: 0 '***' 0.001 '**' 0.01 '*' 0.05 '.' 0.1 ' ' 1

B. Pairwise Post-hoc Comparisons (vs. Control)

Contrast	Estimate	SE	df	t ratio	Pr(>ltl)
Treatment 1 – Treatment 4	0.001	0.000	214	5.23	2.428e-06 ***
Treatment 2 - Treatment 4	0.000	0.000	214	0.76	1.000
Treatment 3 - Treatment 4	0.000	0.000	214	0.38	1.000
Treatment 6 - Treatment 4	0.000	0.000	214	3.55	0.002 **
Treatment 8 - Treatment 4	0.000	0.000	214	0.22	1.000
Treatment 9 - Treatment 4	0.002	0.000	214	13.10	< 2e-16 ***
Treatment 11 - Treatment 4	0.000	0.000	214	1.77	0.312
Treatment 12 - Treatment 4	0.001	0.000	214	5.44	1.033e-06 ***

Signif. codes: 0 '***' 0.001 '**' 0.01 '*' 0.05 '.' 0.1 ' ' 1

Journal of Experimental Biology • Supplementary information

Table S6. Generalized linear model results evaluating the effects of treatment and sex on asymptotic mass in *Gryllodes sigillatus*. ANOVA table (A) showing the significance of treatment and sex on asymptotic mass using a Gamma-distributed GLM with a log link. Pairwise post-hoc comparisons (B) between each treatment and the control (Treatment 4) based on estimated marginal means (EMMs), using treatment-vs-control contrasts and Holm correction for multiple testing. Reported values include coefficient estimates (Estimate), standard errors (SE), degrees of freedom (df), t-ratios, and p-values (Pr(>|t|)).

A. ANOVA Table for Asymptotic mass (a)

Effect	Df	Deviance	Resid. Df	Resid. Dev	F value	Pr(>F)
Treatment	8	5.542	211	1.226	139.394	< 2e-16 ***
Sex	1	0.098	210	1.127	19.758	1.448e-05 ***
Treatment:Sex	8	0.109	202	1.019	2.734	0.00697 **

Signif. codes: 0 '***' 0.001 '**' 0.01 '*' 0.05 '.' 0.1 ' ' 1

B. Pairwise Post-hoc Comparisons (vs. Control)

Contrast	Estimate	SE	df	t ratio	Pr(>ltl)
Treatment 1 - Treatment 4	1.399	0.094	202	14.90	< 2e-16 ***
Treatment 2 - Treatment 4	0.530	0.076	202	7.01	2.423e-10 ***
Treatment 3 - Treatment 4	-0.018	0.065	202	-0.28	1.00000
Treatment 6 - Treatment 4	0.078	0.059	202	1.32	0.75465
Treatment 8 - Treatment 4	-0.281	0.051	202	-5.48	7.590e-07 ***
Treatment 9 - Treatment 4	-0.214	0.059	202	-3.62	0.00186 **
Treatment 11 - Treatment 4	-0.011	0.052	202	-0.21	1.00000
Treatment 12 - Treatment 4	0.052	0.053	202	0.97	0.99509

Signif. codes: 0 '***' 0.001 '**' 0.01 '*' 0.05 '.' 0.1 ' ' 1

Journal of Experimental Biology • Supplementary information

Table S7. Generalized linear model results evaluating the effects of treatment and sex on maximum relative growth rate (k_U) in *Acheta domesticus*. ANOVA table (A) showing the significance of treatment and sex on maximum relative growth rate (k_U) using a Gamma-distributed GLM with a log link. Pairwise post-hoc comparisons (B) between each treatment and the control (Treatment 4) are based on estimated marginal means (EMMs), using treatment-vs-control contrasts with Holm correction for multiple testing. Reported values include coefficient estimates (Estimate), standard errors (SE), degrees of freedom (df), t-ratios, and p-values (Pr(>|t|)).

A. ANOVA Table for Relative Maximum Growth rate (k_U)

Effect	Df	Deviance	Resid. Df	Resid. Dev	F value	Pr(>F)
Treatment	8	5.542	211	1.226	139.394	< 2e-16 ***
Sex	1	0.098	210	1.127	19.758	1.448e-05 ***
Treatment:Sex	8	0.109	202	1.019	2.734	0.00697 **

Signif. codes: 0 '***' 0.001 '**' 0.01 '*' 0.05 '.' 0.1 ' ' 1

B. Pairwise Post-hoc Comparisons (vs. Control), by Sex

Sex	Contrast	Estimate	SE	df	t ratio	Pr(>ltl)
Female	Treatment 1 - Treatment 4	1.399	0.094	202	14.90	< 2e-16 ***
Female	Treatment 2 - Treatment 4	0.530	0.076	202	7.01	2.423e-10 ***
Female	Treatment 3 - Treatment 4	-0.018	0.065	202	-0.28	1.00000
Female	Treatment 6 - Treatment 4	0.078	0.059	202	1.32	0.75465
Female	Treatment 8 - Treatment 4	-0.281	0.051	202	-5.48	7.590e-07 ***
Female	Treatment 9 - Treatment 4	-0.214	0.059	202	-3.62	0.00186 **
Female	Treatment 11 - Treatment 4	-0.011	0.052	202	-0.21	1.00000
Female	Treatment 12 - Treatment 4	0.052	0.053	202	0.97	0.99509
Male	Treatment 1 - Treatment 4	1.602	0.091	202	17.53	< 2e-16 ***
Male	Treatment 2 - Treatment 4	0.797	0.071	202	11.18	< 2e-16 ***
Male	Treatment 3 - Treatment 4	0.046	0.055	202	0.83	0.81154
Male	Treatment 6 - Treatment 4	0.256	0.066	202	3.87	8.941e-04 ***
Male	Treatment 8 - Treatment 4	-0.119	0.060	202	-1.98	0.24290
Male	Treatment 9 - Treatment 4	0.085	0.065	202	1.30	0.58533
Male	Treatment 11 - Treatment 4	-0.015	0.066	202	-0.23	0.81955
Male	Treatment 12 - Treatment 4	0.108	0.064	202	1.70	0.36271

Signif. codes: 0 '***' 0.001 '**' 0.01 '*' 0.05 '.' 0.1 ' ' 1

Journal of Experimental Biology • Supplementary information

Table S8. Generalized linear model results evaluating the effects of treatment and sex on maximum relative growth rate (k_U) in *Gryllodes sigillatus*. ANOVA table (A) showing the significance of treatment and sex on maximum relative growth rate (k_U) using a Gamma-distributed GLM with a log link. Pairwise post-hoc comparisons (B) between each treatment and the control (Treatment 4) are based on estimated marginal means (EMMs), using treatment-vs-control contrasts with Holm correction for multiple testing. Reported values include coefficient estimates (Estimate), standard errors (SE), degrees of freedom (df), t-ratios, and p-values (Pr(>|t|)).

A. ANOVA Table for Maximum Growth rate (k_U)

Effect	Df	Deviance	Resid. Df	Resid. Dev	F value	Pr(>F)
Treatment	11	7.901	313	3.034	84.624	< 2e-16 ***
Sex	1	0.192	312	2.841	22.677	2.983e-06 ***
Treatment:Sex	11	0.260	301	2.581	2.784	0.00183 **

Signif. codes: 0 '***' 0.001 '**' 0.01 '*' 0.05 '.' 0.1 ' ' 1

B. Pairwise Post-hoc Comparisons (vs. Control), by Sex

Sex	Contrast	Estimate	SE	df	t ratio	Pr(>ltl)
Female	Treatment 1 - Treatment 4	2.215	0.172	301	12.85	< 2e-16 ***
Female	Treatment 2 - Treatment 4	0.460	0.084	301	5.47	9.448e-07 ***
Female	Treatment 3 - Treatment 4	-0.079	0.073	301	-1.08	1.00000
Female	Treatment 5 - Treatment 4	0.122	0.089	301	1.36	1.00000
Female	Treatment 6 - Treatment 4	0.102	0.089	301	1.15	1.00000
Female	Treatment 7 - Treatment 4	0.102	0.099	301	1.02	1.00000
Female	Treatment 8 - Treatment 4	-0.333	0.073	301	-4.55	6.874e-05 ***
Female	Treatment 9 - Treatment 4	-0.321	0.072	301	-4.44	9.952e-05 ***
Female	Treatment 10 - Treatment 4	0.014	0.102	301	0.14	1.00000
Female	Treatment 11 - Treatment 4	0.058	0.082	301	0.71	1.00000
Female	Treatment 12 - Treatment 4	0.050	0.083	301	0.60	1.00000
Male	Treatment 1 - Treatment 4	2.052	0.121	301	17.00	< 2e-16 ***
Male	Treatment 2 - Treatment 4	0.479	0.090	301	5.31	2.137e-06 ***
Male	Treatment 3 - Treatment 4	0.012	0.065	301	0.19	1.00000
Male	Treatment 5 - Treatment 4	0.011	0.072	301	0.16	1.00000
Male	Treatment 6 - Treatment 4	0.106	0.071	301	1.49	0.67989
Male	Treatment 7 - Treatment 4	0.378	0.081	301	4.69	3.343e-05 ***

Sex	Contrast	Estimate	SE	df	t ratio	Pr(>ltl)
Male	Treatment 8 - Treatment 4	-0.237	0.070	301	-3.38	0.00574 **
Male	Treatment 9 - Treatment 4	-0.134	0.074	301	-1.82	0.42242
Male	Treatment 10 - Treatment 4	0.547	0.110	301	4.98	9.753e-06 ***
Male	Treatment 11 - Treatment 4	-0.086	0.082	301	-1.05	0.88932
Male	Treatment 12 - Treatment 4	0.105	0.077	301	1.37	0.68771

Signif. codes: 0 ‘***’ 0.001 ‘**’ 0.01 ‘*’ 0.05 ‘.’ 0.1 ‘ ’ 1

Table S9. Summary of generalized linear model results evaluating the effects of treatment and sex on the slope of development in *Acheta domesticus*. ANOVA table (A) showing the significance of treatment and sex using a Gamma-distributed GLM with a log link. Pairwise post-hoc comparisons (B) between each treatment and the control (Treatment 4) are based on estimated marginal means (EMMs), using treatment-vs-control contrasts with Holm correction for multiple testing. Reported values include coefficient estimates (Estimate), standard errors (SE), degrees of freedom (df), t-ratios, and p-values (Pr(>|t|)).

A. ANOVA Table for Slope of Development

Effect	Df	Deviance	Resid. Df	Resid. Dev	F value	Pr(>F)
Treatment	8	5.412	222	1.710	91.870	< 2e-16 ***
Sex	1	0.079	221	1.631	10.737	0.00122 **

Signif. codes: 0 ‘***’ 0.001 ‘**’ 0.01 ‘*’ 0.05 ‘.’ 0.1 ‘ ’ 1

B. Pairwise Post-hoc Comparisons (vs. Control)

Contrast	Estimate	SE	df	t ratio	Pr(>ltl)
Treatment 1 - Treatment 4	0.387	0.022	221	17.76	< 2e-16 ***
Treatment 2 - Treatment 4	0.195	0.018	221	11.04	< 2e-16 ***
Treatment 3 - Treatment 4	0.016	0.014	221	1.15	0.72319
Treatment 6 - Treatment 4	0.048	0.015	221	3.14	0.00974 **
Treatment 8 - Treatment 4	-0.082	0.013	221	-6.23	1.392e-08 ***
Treatment 9 - Treatment 4	0.019	0.016	221	1.18	0.72319
Treatment 11 - Treatment 4	-0.000	0.014	221	-0.02	0.98187
Treatment 12 - Treatment 4	0.034	0.014	221	2.43	0.06375 .

Signif. codes: 0 ‘***’ 0.001 ‘**’ 0.01 ‘*’ 0.05 ‘.’ 0.1 ‘ ’ 1

Table S10. Generalized linear model results evaluating the effects of treatment and sex on the slope of development in *Gryllodes sigillatus*. ANOVA table (A) showing the significance of treatment, sex and their interaction using a Gamma-distributed GLM with a log link. Pairwise post-hoc comparisons (B) between each treatment and the control (Treatment 4) are based on estimated marginal means (EMMs), using treatment-vs-control contrasts with Holm correction for multiple testing. Reported values include coefficient estimates (Estimate), standard errors (SE), degrees of freedom (df), t-ratios, and p-values (Pr(>|t|)).

A. ANOVA Table for Slope of Development

Effect	Df	Deviance	Resid. Df	Resid. Dev	F value	Pr(>F)
Treatment	11	7.565	319	3.226	74.230	< 2e-16 ***
Sex	1	0.025	318	3.202	2.681	0.10256
Treatment:Sex	11	0.239	307	2.963	2.344	0.00876 **

Signif. codes: 0 ‘***’ 0.001 ‘**’ 0.01 ‘*’ 0.05 ‘.’ 0.1 ‘ ’ 1

B. Pairwise Post-hoc Comparisons (vs. Control), by Sex

Sex	Contrast	Estimate	SE	df	t ratio	Pr(>ltl)
Female	Treatment 1 - Treatment 4	0.509	0.043	307	11.93	< 2e-16 ***
Female	Treatment 2 - Treatment 4	0.101	0.023	307	4.49	9.049e-05 ***
Female	Treatment 3 - Treatment 4	-0.010	0.020	307	-0.50	1.00000
Female	Treatment 5 - Treatment 4	0.038	0.025	307	1.52	0.90482
Female	Treatment 6 - Treatment 4	0.020	0.025	307	0.80	1.00000
Female	Treatment 7 - Treatment 4	0.030	0.027	307	1.12	1.00000
Female	Treatment 8 - Treatment 4	-0.120	0.020	307	-6.11	3.047e-08 ***
Female	Treatment 9 - Treatment 4	-0.086	0.020	307	-4.21	2.691e-04 ***
Female	Treatment 10 - Treatment 4	0.022	0.031	307	0.72	1.00000
Female	Treatment 11 - Treatment 4	0.007	0.022	307	0.31	1.00000
Female	Treatment 12 - Treatment 4	0.016	0.022	307	0.72	1.00000
Male	Treatment 1 - Treatment 4	0.499	0.034	307	14.68	< 2e-16 ***
Male	Treatment 2 - Treatment 4	0.120	0.027	307	4.52	6.926e-05 ***
Male	Treatment 3 - Treatment 4	-0.003	0.019	307	-0.18	1.00000
Male	Treatment 5 - Treatment 4	-0.020	0.021	307	-0.97	1.00000
Male	Treatment 6 - Treatment 4	0.024	0.021	307	1.16	0.99157
Male	Treatment 7 - Treatment 4	0.087	0.024	307	3.58	0.00280 **

Journal of Experimental Biology • Supplementary information

Sex	Contrast	Estimate	SE	df	t ratio	Pr(>ltl)
Male	Treatment 8 - Treatment 4	-0.111	0.021	307	-5.42	1.209e-06 ***
Male	Treatment 9 - Treatment 4	-0.035	0.024	307	-1.48	0.69972
Male	Treatment 10 - Treatment 4	0.183	0.036	307	5.06	6.471e-06 ***
Male	Treatment 11 - Treatment 4	0.009	0.024	307	0.38	1.00000
Male	Treatment 12 - Treatment 4	0.050	0.023	307	2.15	0.19512

Signif. codes: 0 ‘***’ 0.001 ‘**’ 0.01 ‘*’ 0.05 ‘.’ 0.1 ‘ ’ 1

Journal of Experimental Biology • Supplementary information

Table S11. Generalized linear model results evaluating the effect of treatment on developmental performance in *Acheta domesticus*. ANOVA table (A) showing the significance of treatment using a Gamma-distributed GLM with a log link. Pairwise post-hoc comparisons (B) between each treatment and the control (Treatment 4) are based on estimated marginal means (EMMs), using treatment-vs-control contrasts with Holm correction for multiple testing. Reported values include coefficient estimates (Estimate), standard errors (SE), degrees of freedom (df), t-ratios, and p-values (Pr(>|t|)).

A. ANOVA Table for Developmental Performance

Effect	Df	Deviance	Resid. Df	Resid. Dev	F value	Pr(>F)
Treatment	8	35.225	222	11.419	80.406	< 2e-16 ***

Signif. codes: 0 '***' 0.001 '**' 0.01 '*' 0.05 '.' 0.1 ' ' 1

B. Pairwise Post-hoc Comparisons (vs. Control)

Contrast	Estimate	SE	df	t ratio	Pr(>ltl)
Treatment 1 - Treatment 4	0.965	0.119	222	8.10	2.551e-13 ***
Treatment 2 - Treatment 4	0.561	0.095	222	5.93	6.834e-08 ***
Treatment 3 - Treatment 4	-0.004	0.068	222	-0.06	1.00000
Treatment 6 - Treatment 4	0.436	0.089	222	4.88	9.915e-06 ***
Treatment 8 - Treatment 4	-0.045	0.068	222	-0.66	1.00000
Treatment 9 - Treatment 4	4.864	0.334	222	14.55	< 2e-16 ***
Treatment 11 - Treatment 4	-0.041	0.066	222	-0.63	1.00000
Treatment 12 - Treatment 4	0.214	0.074	222	2.88	0.01756 *

Signif. codes: 0 '***' 0.001 '**' 0.01 '*' 0.05 '.' 0.1 ' ' 1

Table S12. Generalized linear model results evaluating the effect of treatment on developmental performance in *Gryllodes sigillatus*. ANOVA table (A) showing the significance of treatment using a Gamma-distributed GLM with a log link. Pairwise post-hoc comparisons (B) between each treatment and the control (Treatment 4) are based on estimated marginal means (EMMs), using treatment-vs-control contrasts with Holm correction for multiple testing. Reported values include coefficient estimates (Estimate), standard errors (SE), degrees of freedom (df), t-ratios, and p-values (Pr(>|t|)).

A. ANOVA Table for Developmental Performance

Effect	Df	Deviance	Resid. Df	Resid. Dev	F value	Pr(>F)
Treatment	11	57.182	319	22.992	71.548	< 2e-16 ***

Signif. codes: 0 '***' 0.001 '**' 0.01 '*' 0.05 '.' 0.1 ' ' 1

B. Pairwise Post-hoc Comparisons (vs. Control)

Contrast	Estimate	SE	df	t ratio	Pr(>ltl)
Treatment 1 - Treatment 4	2.696	0.223	319	12.11	< 2e-16 ***
Treatment 2 - Treatment 4	0.297	0.078	319	3.81	8.424e-04 ***
Treatment 3 - Treatment 4	0.117	0.063	319	1.86	0.19227
Treatment 5 - Treatment 4	0.131	0.073	319	1.80	0.19227
Treatment 6 - Treatment 4	0.218	0.075	319	2.92	0.01485 *
Treatment 7 - Treatment 4	0.661	0.105	319	6.28	9.855e-09 ***
Treatment 8 - Treatment 4	-0.226	0.059	319	-3.85	8.424e-04 ***
Treatment 9 - Treatment 4	-0.030	0.068	319	-0.45	0.65635
Treatment 10 - Treatment 4	7.728	0.682	319	11.33	< 2e-16 ***
Treatment 11 - Treatment 4	0.355	0.085	319	4.18	2.667e-04 ***
Treatment 12 - Treatment 4	0.453	0.086	319	5.28	1.943e-06 ***

Signif. codes: 0 '***' 0.001 '**' 0.01 '*' 0.05 '.' 0.1 ' ' 1

Journal of Experimental Biology • Supplementary information
